# A Systematic Review on Pulmonary Complications Secondary to Hematopoietic Stem Cell Transplantation

**DOI:** 10.7759/cureus.24807

**Published:** 2022-05-07

**Authors:** Alberto Busmail, Sai Sri Penumetcha, Saher Ahluwalia, Rejja Irfan, Sawleha Arshi Khan, Sai Rohit Reddy, Maria Elisa Vasquez Lopez, Maryam Zahid, Lubna Mohammed

**Affiliations:** 1 Internal Medicine, California Institute of Behavioral Neurosciences & Psychology, Fairfield, USA; 2 Research, California Institute of Behavioral Neurosciences & Psychology, Fairfield, USA; 3 School of Medicine, Armed Forces Medical College, Pune, IND; 4 Research & Development, California Institute of Behavioral Neurosciences & Psychology, Fairfield, USA

**Keywords:** obliterative bronchiolitis, hematopoietic stem cell transplant, pleuroparenchymal fibroelastosis, diffuse alveolar hemorrhage, key words– pulmonary hypertension

## Abstract

The main purpose of this systematic review was to identify and synthesize evidence about pulmonary complications following stem cell transplantation to raise awareness among physicians since it is a lesser-known topic. Studies that included targeted pulmonary complications that occurred after stem cell transplantation; in humans; and were randomized controlled trials, cohort studies, and case studies between January 2011 and 2021. Fifteen intervention features were identified and analyzed in terms of their association with successful or unsuccessful interventions. Fifteen of 15 studies that met inclusion criteria had positive results. Features that appeared to have the most consistent positive effects included relevant information consisting of clinical presentations and management of complications.

Hematopoietic stem cell transplantation is a therapeutic method that has been introduced for various hematological diseases. Its main objective is to restore the hematopoietic function that has been eradicated or affected. The stem cell transplantation requires a period of administration of chemotherapeutic agents that may lead to infectious and/or non-infectious pulmonary complications that require follow-up.

Noninfectious pulmonary complications include bronchiolitis obliterans, alveolar hemorrhage, fibroelastosis, pulmonary hypertension, and infections. Bronchiolitis obliterans syndrome is an obstructive lung disease that affects the small airways, reducing lung function, and it’s the most frequent late-onset complication. Furthermore, diffuse pulmonary hemorrhage is a fatal adverse effect and the most common noninfectious pulmonary complication of acute leukemia, observed within the first weeks after the procedure. Pulmonary hypertension has multiple etiologies, mainly related to the pulmonary veno-occlusive disease. It carries a poor prognosis, with a 55% mortality rate. The area of hematology is very wide and prone to new development of treatments and procedures that could be available for new emerging diseases and improving survival rates.

## Introduction and background

Over the decades, stem cell transplantation (STC) has been modified and introduced as therapy management for various malignant and nonmalignant hematological diseases and has now emerged to improve the prognosis of hematopoietic diseases. There are various types of transplants -syngeneic, autologous, and allogenic - differing in their technique, donor source, complications, and incidence range. In a syngeneic transplant, the stem cells derive from a genetically identical donor (twin); in an autologous transplant, the stem cells come from the patient, and in an allogenic transplant, the stem cells originate from a matching donor. The stem cells can be obtained from the donor’s bone marrow, peripheral blood, or cord blood. The main function of STC is to restore the hematopoietic function that has been eradicated by the disease or chemotherapy, which destroys the malignant cells and inevitably, the stem cells [1]. Stem cells are administered intravenously to regenerate the bone marrow function, and post-transplant immunosuppressive therapy must be given [1,2].

Transplant-related complications are sub-classified based on their time of onset: neutropenic phase (1-30 days), early post-engraftment phase (30-100 days), and late post-engraftment phase (beyond 100 days) [3,4]. Despite its advances in therapy management and transplant techniques, stem cell transplantation is associated with life-threatening side effects, constituting more than half of non-infectious complications in pulmonary pathology [4,5].

Management of hematologic malignancies places the patient at various disadvantages, such as an immunocompromised state which makes them more susceptible to infections and non-infectious complications. About 20% of allogenic STC recipients develop a late pulmonary complication, and its success depends on acute and chronic pulmonary disease [2]. Pulmonary complications are the primary cause of mortality and treatment-related morbidity following STC, responsible for 40-60% of pulmonary cases [6]. The mortality rate is associated with certain risk factors: male sex, history of pulmonary disease, and disease condition at the time of transplant, which determines the outcome of survival [1].

Noninfectious pulmonary complications include bronchiolitis obliterans, hemorrhage, and pulmonary hypertension [2]. Clinically, these patients present a vast array of manifestations, making it challenging to diagnose these complications, which materialize with dyspnea, fever, nonproductive cough, and hypoxia within the first few weeks of the transplant.

Bronchiolitis obliterans syndrome - the most feared complication- is a potentially life-threatening side effect defined as an airflow obstruction that affects 1%-2% of allogenic stem cell transplantation (SCT) recipients, usually during the first two years being difficult to diagnose due to its nonspecific symptoms [7,8]. Pulmonary infections - bacterial, fungal, and viral - are responsible for 50% of all pulmonary complications after SCT, being the main cause of death in these patients, due to its complex therapy management and the immunosuppression of the patient, who tend to get infected during the neutropenic phase and after chemotherapy [5]. Uncommon complications are now emerging due to the use of new drugs in therapy management.

Although this topic has been heavily researched, it is still poorly known by physicians, especially the pathogenesis of each complication, making the goal of this systematic review to educate clinicians about the pulmonary complications that may arise following a bone marrow transplant, being an uncommon topic.

Methods

This systematic review was designed following criteria set by the Preferred Reporting Items for Systematic Reviews and Meta-Analyses (PRISMA) statement [9].

Search Strategy

For this systematic review, two databases, PubMed/PubMed Central and Google Scholar were thoroughly searched using the most relevant keywords and Medical Subject Headings (MeSH) concepts to retrieve the most significant articles demonstrating pulmonary complications following bone marrow transplant. The keywords used include bone marrow transplant, hematopoietic transplant, stem cell transplant, and pulmonary complications. The Boolean scheme was used as well to combine keywords and the MeSH strategy format, which was later used in PubMed. The articles acquired were thoroughly revised to obtain the most relevant studies and information.

Inclusion and Exclusion Criteria

The exclusion criteria are as follows: (1) articles must include information on pulmonary complications after bone marrow transplantation; (2) reviewed articles and studies for this systematic review need to be published between 2011 and 2021; (3) studies describing pulmonary complications following bone marrow transplant must be conducted in humans; and (4) all articles used were published in English.

Data Extraction

Data selection and extraction were approved by two researchers independently.

Analysis of Study Quality

The cohort retrospective and case-control studies were analyzed with the New Castle Ottawa criteria; meanwhile, the quality of the systematic review was assessed with the Preferred Reporting Items for Systematic Reviews.

Results

**Figure 1:**
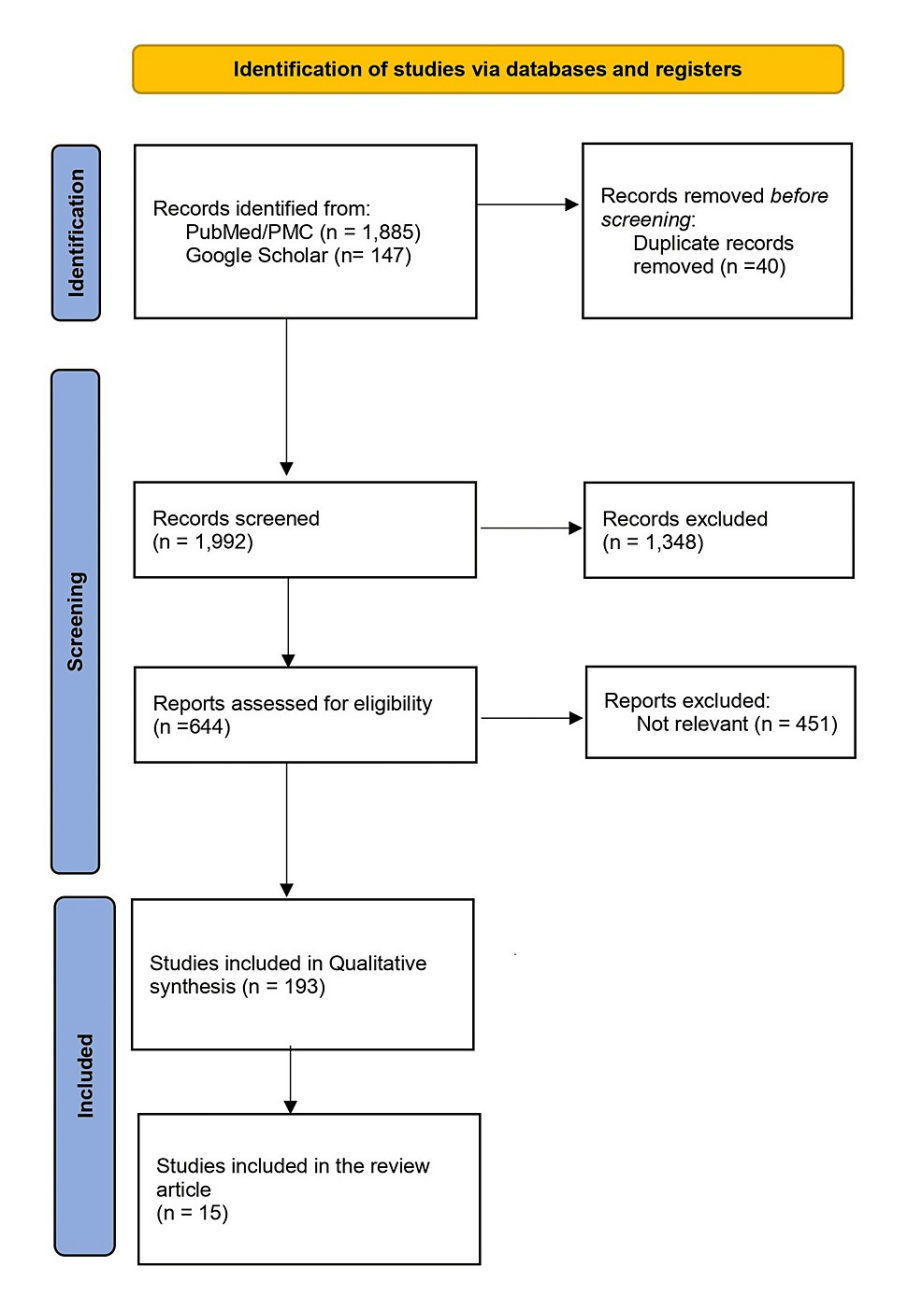
Identification of studies via databases and registers

The online search yielded 2,032 articles from two different databases, out of which only 40 articles were duplicates and subsequently removed, leaving 1,992 to be screened. Using the inclusion/exclusion criteria: must include pulmonary complications after bone marrow transplant, human studies published between 2011 and 2021, and the English language, a total of 1,348 were removed. Afterward, 644 articles were assessed, and only 193 articles were relevant to the study question. A total of 15 articles fulfilled the qualitative criteria and PRISMA checklist for the study design, and the search processes are illustrated in Figure 1.

## Review

Pulmonary complications secondary to stem cell transplantation cause severe problems in hematopoietic patients. The risks represent a high mortality rate with difficult multifactorial manifestations to treat. These side effects cause non-infectious and infectious complications that are greatly associated with several risk factors. The most common non-infectious pulmonary complications are bronchiolitis obliterans syndrome, alveolar hemorrhage, fibroelastosis, pulmonary hypertension, air leak syndrome, interstitial lung disease, and infections. These complications are the primary cause of mortality related to stem cell transplantation, reducing the patient’s lung function and survival rate. 

Bronchiolitis obliterans

Bronchiolitis obliterans syndrome (BOS) is an obstructive lung disease that affects the small airways, reducing lung function over time. It is the most frequent late-onset noninfectious pulmonary complication, affecting 4%-9% of pediatric SCT recipients and 5.5% of adult SCT recipients [10]. Its etiology is unknown, but studies suggest there is evidence that viral infections induced by T-cells cause inflammation, leading to fibrosis, playing an important role in its pathogenesis [11]. Several factors such as chemotherapy, radiotherapy, gastroesophageal reflux, and respiratory pathogens cause a bronchiolar epithelium injury followed by an inflammatory reaction, causing fibrosis over time and obstruction of small airways. BOS has a vast clinical course, various manifestations, and different triggers, which indicate a multifactorial physiopathology. BOS presents nonspecific symptoms which vary between patients, making it hard to diagnose. Patients manifest cough, dyspnea, wheezing, and repeated lung infections but may be asymptomatic during the early stages. Deaths related to BOS are associated with respiratory failure or infections due to the immunosuppressed state of the patient [11,12].

A cross-sectional case-control study was performed in a pediatric population 3-10 years after SCT to evaluate survival rate, including 64 patients and 64 matched controls. [10] The following parameters were considered: age at the time of transplant, primary diagnosis, type of donor, graft source, donor age, and sex.

Multiple breath nitrogen washout (MBWN2) has been used in pediatric survivors of SCT. This is a sensitive method to evaluate small airway function. The study reported that small airway dysfunction measured by MBWN2 was a common finding in the pediatric population after allogenic SCT with no respiratory symptoms. MBWN2 is used as an early marker to evaluate long-term consequences of signs of small airway dysfunction [10].

There is no curative treatment for BOS since it’s a pulmonary complication. Its therapeutic treatment is mainly focused on preventing further lung function deterioration [11]. Extracorporeal photopheresis (ECP) is a common treatment and was used on patients with severe bronchiolitis obliterans syndrome to evaluate over a long period the forced expiratory volume in one second (FEV1). In this study, eight patients developed BOS after SCT and a decline in FEV1 despite receiving steroid therapy. The patients were submitted to ECP therapy for one year to further evaluate the FEV1. Over one year, two of the eight patients had a stable FEV1, decreasing the rate of decline in FEV1 with the use of ECP. All patients survived the first year of ECP therapy, resulting in a decline in the dose of prednisone per patient. It was concluded that ECP shows a beneficial rate of decline in FEV1 in pulmonary disease [13].

Systemic steroids are considered the main therapy, although there’s a lack of evidence regarding its efficacy and multiple side effects. Antifibrotic medication is being considered for BOS treatment due to its effectiveness in slowing idiopathic pulmonary fibrosis [11]. 

Alveolar hemorrhage

Diffuse pulmonary hemorrhage is fatal and the most common, noninfectious pulmonary complication of acute leukemia, observed in 20% of patients after SCT within the first weeks after the procedure. Its mortality incidence rate varies between 64%-100% due to multiorgan failure or sepsis, despite the use of intensive care units and mechanical ventilation [14-16]. Its physiopathology is multifactorial and is associated with inflammation, lung tissue injury produced by high dose total body irradiation, graft source, cytokine release, delayed engraftment, or graft failure; these are all considered to play a role in its pathogenesis. A study done on pediatric and adolescent patients reported that pulmonary hemorrhage occurred in 125 children 100 days after the hematopoietic cell transplantation (HCT). These patients have a poor prognosis rate, with only 23% surviving 100 days after developing alveolar hemorrhage and 16% surviving up to six months [16,17]. The University of Minnesota conducted a study with 1228 patients who underwent SCT between 2008-2015 to analyze the incidence, risk factors, and outcomes of diffuse alveolar hemorrhage. Alveolar hemorrhage was developed in 5% of allogenic SCT recipients, with a medium of 30 days after the procedure. Recipients of umbilical cord blood had a greater incidence rate than those of peripheral blood or marrow grafts. Patients with diffuse alveolar hemorrhage had an inferior six-month treatment-related mortality and a two-year overall survival using either graft source [14].

Clinically, patients present dyspnea, nonproductive cough, fever, hypoxia, and in rare cases, hemoptysis. Chest X-rays report diffuse pulmonary infiltrates and opacity in lower lung zones [15]. These radiographic changes rapidly evolve into diffuse ground-glass opacities and consolidation. Its treatment includes the use of steroids, platelet transfusion, antifibrinolytic, and pro-coagulant therapies, but there’s not enough evidence to suggest the beneficial use of these treatments. Clinical interventions and experimental trials have been implemented to reduce the risk factors and improve the survival rate of these patients [16,17].

Fibroelastosis

Pleuroparenchymal fibroelastosis (PPFE) is a late-onset noninfectious pulmonary complication that occurs after SCT. It’s characterized by intense elastic fibrosis in the pleura and subpleural lung parenchyma in the upper lobes. Its pathogenesis is unknown, but it’s associated with cytotoxic agents (especially alkylating agents used in hematological diseases) [18]. The chronic graft-versus-host disease (cGVHD), in which the recipient’s body attacks the donated cells, is also considered a possible cause of its pathogenesis [19]. PPF has also been noted in cases of autologous SCT, chemotherapy, and lung transplantation. Several studies have been analyzed following PPFE after allogeneic SCT to investigate the etiology. A case report was presented of a 31-year-old patient who underwent an allogenic bone marrow transplant, developing PPFE abnormalities as a late-onset noninfectious pulmonary complication strongly associated with cGVHD. The patient presented dyspnea, a nonproductive cough for six months, and a history of allogenic bone marrow transplants nine years ago [19]. Chest X-rays showed pleural and subpleural fibrosis with upper lobe predominance, small bilateral pneumothoraces, and a deteriorated upper lobe despite the treatment with inhaled and systemic corticosteroids [19]. PPFE has a poor prognosis and has no definitive treatment, but antifibrotic drugs are used despite the lack of evidence of beneficial effects [16].

Pulmonary hypertension

Pulmonary hypertension (PH) has multiple causes and is a known complication secondary to STC. Its incidence is unknown, but 40 cases have been reported, primarily in the pediatric population. PH has a poor prognosis, with a 55% mortality rate. The etiology of post SCT PH is elusive, but it’s mainly related to pulmonary veno-occlusive disease [16]. PH is hard to identify due to its vast symptomatology, and it’s usually diagnosed when it is severe and symptomatic [20]. A study was done at the City of Hope Medical Center that included 60 patients who underwent STC from 2008 to April 2018. Patients’ age, sex, race, diagnosis, and comorbidity index were all taken into consideration. The study concluded that 57% developed PH; they were older than those who did not have PH and that PH is common in patients who undergo STC [20].

Air leak syndrome

Air leak syndrome (ALS) is another rare pulmonary complication post allogenic SCT, characterized by all forms of thoracic air leakage: pneumothorax, pneumopericardium, pneumomediastinum, and subcutaneous emphysema. Its incidence is 0.8%-2.3%, with a poor prognosis and a mortality rate of 10%-40% in patients [21]. Its pathogenesis is unknown, but several causes induce these patients to develop ALS. Due to the hematologic disease, the drug treatments taken can induce pulmonary injuries, pulmonary edema, and interstitial pneumonia, causing a more fragile alveolar wall and decreased lung compliance, making patients more vulnerable to developing ALS [22,23]. Clinically, patients present cough, dyspnea, chest pain, or chest tightness, and radiological abnormalities 100 days post stem cell transplantation with no apparent infectious cause [23]. These symptoms are caused by lung tissue damage, inflammatory reactions, and the pressure of free air in the cardiopulmonary vessels [22].

Several risk factors are associated with ALS, most commonly young age, male sex, multiple SCT, and cGVHD. Between January 2003 and December 2014, 423 patients that received allogenic SCT were analyzed to determine the most common characteristics associated with ALS. The median age was 33 years, predominantly in males, with a follow-up of 19.8 months and an increase in pulmonary invasive fungal infections [23]. The median onset was 68.8 months after bone marrow transplantation, manifesting progressive dyspnea, sudden chest pain, or fever.

All patients with ALS had pneumothorax, one patient having pneumomediastinum and subcutaneous emphysema. The radiological findings tend to be similar in all patients, presenting subpleural thickening in the upper lung fields, glass opacification, and bronchiectasis in the upper lobes. Its diagnosis can be guided through radiological imaging, computed tomography (CT) (the standard method), and pathologically through histological findings.

A study from January 2014 to December 2018 was conducted at the Institute of Hematology and Blood Diseases Hospital, Chinese Academy of Medical Sciences, and Peking Union Medical College to analyze clinical and imaging characteristics. They retrospectively analyzed age, sex, height, weight, hematologic disease, numbers of basic treatments, symptoms, type of lung disease, inducing factors, history of allogenic SCT, and types of ALS [22]. The study reported that ALS has a high rate in young males with low BMI (body mass index), with a male ratio of 68.8% and a mean BMI of 19.4. The main inducing factor was a cough, with 62.5% in patients with a history of allogenic SCT. The most common symptoms were dyspnea at 31.3% and chest tightness at 68.8%. Conservative treatment is given in patients complicated with ALS. Supplementary oxygen, rest, and antibiotics are commonly used, and in severe cases, catheter drainage is required.

Interstitial lung disease

Interstitial lung disease (ILD) is defined as the presence of diffuse lung parenchymal opacities on a scan, usually misdiagnosed due to scarce information and combined treatment. Its pathogenesis is elusive, but medical exposure and collagen vascular diseases are attributed to its cause.

Between 2001 and 2010, 40 patients were included in the study to analyze clinical characteristics, pulmonary function tests, radiological features, and outcomes of patients with allogenic SCT diagnosed with ILD with a median time of 11.3 months. In 75% of cases, peripheral blood was used, out of whom 30% had a history of autologous transplantation. The most common symptom was dyspnea, with an onset of 15 days. Its diagnosis is based on clinical characterization, high-resolution computed tomography, pulmonary function testing, and lung histology. In the CT, two patterns were identified: ground-glass opacities or alveolar consolidations. The treatment was focused on 35 (87.5%) patients receiving systemic steroids and 13 of these high-dose intravenous steroids. Thirteen patients died (32.5%), of which 10 were due to respiratory failure. The prognostic analysis was based on patients with alveolar consolidation tending to have a better survival rate than those with ground-glass opacity, but the difference rate was not taken into statistical significance [24].

Infections

Pulmonary complications are the complications with the highest mortality rate in patients, with 32% of all cases [25,26] secondary to SCT. Their immunosuppressive state makes them vulnerable to infections, worsening their overall health and survival rate. Early identification of infection and microorganisms contributes to improving the patient’s chances of survival. It was identified that pneumonia was a common complication in immunocompromised hosts [25]. A cohort study of hematopoietic recipients was done to identify risk factors, etiology, diagnostic procedures, and outcome of the pulmonary infections. From the 169 patients, 73 pulmonary complications were identified: 50 (68%) were pneumonia.

The etiology of the pneumonia cases were viruses (Rhinovirus) and bacteria (*P. aeruginosa*). A total of 50 cases of pneumonia were identified, mostly viral (28%), bacterial (26%), and fungal (16%). Combined infections, both viral and bacterial, were identified in five episodes (10%). The most common viral causes were Rhinovirus and respiratory syncytial virus. *Pseudomonas aeruginosa* was the most common microorganism, representing 61% of all bacterial infections. Its diagnosis is based on fiberoptic bronchoscopy (FOB) and non-invasive explorations. The diagnostic procedures strictly depend on clinical judgment and empirical treatment. If there’s no response to the empirical treatment, computed tomography is indicated, FOB, bronchial aspiration in pulmonary diffuse infiltrates, and specific primers in RT-PCR in nasopharyngeal swabs were used for the detection of viruses [25,27,28].

The department of Pediatrics at National Taiwan University Hospital investigated the outcome and the most common pathogen in immunocompromised hosts [27]. The viremia caused by the cytomegalovirus was identified through a real-time polymerase chain reaction (RT-PCR) at a median of 23 days post SCT. Four patients presented complications: colitis, pneumonitis complicated with pulmonary hemorrhage, and sepsis. Several risk factors were associated with greater complications, such as older diagnostic age, leukemic patients, and unrelated donors that contribute to a high mortality rate [27]. CMV viremia has a high incidence, 32%, due to its severe complications, which is why a strict observation can reduce morbidity and mortality rates [27].

Predictors of mortality

Pulmonary complications have a poor prognosis in children due to severe complications and the need for mechanical ventilation. A retrospective observational study was done to evaluate the early predictors of mortality in children with pulmonary complications after the hematopoietic stem cell transplantation. The focus of the study was centered on the diagnostic procedures and work-ups, therapy modification, and medical conditions to predict mortality. The study collected data from January 2011 and December 2012, including age, sex, previous lung problems, type and number of SCT, primary disease, and stem cell source. A pulmonary complication was characterized as a new pulmonary infiltrate on a chest X-ray accompanied by respiratory symptoms. These symptoms included: dyspnea, cough, sputum, chest pain, hemoptysis, and cyanosis. The diagnosis is established through radiologic, microbiologic, and pathological tests [29].

The diagnostic evaluations were based on using invasive procedures, such as bronchoalveolar lavage, and non-invasive tests: blood cultures, sputum examination, serology tests, chest CT scans, echocardiography, and pulmonary function tests. All exams were used to identify infections and different pathogens. The patients’ characteristics reported 35 recipients (25 males and 10 females) with one pulmonary infiltrate secondary to SCT, with a median age of 10.5 years. The majority of the patients had leukemia and lymphoma (60%), and 45.7% had a lung problem before SCT. Sixty percent of the transplantations were allogenic and originated from peripheral blood. Early predictors of mortality associated with poor outcomes were hematologic (neutropenia) and hepatic dysfunction [30]. Complications following SCT can cause respiratory failure with mechanical ventilation and single or multiple organ failure in response to chemotherapy, and inflammatory response leading to a pro-coagulant state. Organ dysfunction of the central nervous system, liver, and lungs showed a poor outcome, using extracorporeal organ support in critical patients. The mortality factors make patients vulnerable to different complications, requiring supportive care and worsening their prognosis [30]. Overview and characteristics of included studies that expose the pulmonary complications following SCT are presented in the summary table (Table 1).

**Table 1:** Overview of the eligible studies examining the pulmonary complications following hematopoietic stem cell transplantation

Studies Used in this Systematic Review					
Author, year	Study design	Location	Sample Size	Mean patient age	Mean follow-up
Uhlving HH, 2014 [9]	Cross-sectional case control study	Denmark	64 patients	N/A	N/A
Brownback KR, 2015 [12]	Prospective cohort study	United States	8 patients	43.5 years old	1 year
Keklik F, 2018 [13]	Prospective cohort study	United States	1228 patients	32 years	N/A
Broglie Ll, 2019 [16]	Case control	United States	5022 patients	8 years old	6 years
Fujikura Y, 2014 [18]	Case report	Japan	1 patient	31 years old	N/A
Gupta R, 2019 [19]	Retrospective analysis-case control	United States	65 patients	61 years old	35 months
Ishii T, 2016 [20]	Retrospective analysis-case control	Japan	5 patients	37 years	68.8 months
Liu Y-C, 2019 [22]	Retrospective cohort study	Taiwan	423 patients	42 years	1 year
Schlemmer F, 2014 [23]	Retrospective study	France	40 patients	40.5 years of age	9 months
Lucena CM, 2014 [24]	Prospective cohort study	Spain	169 patients	49 years	1 year
Pipavath SNJ, 2012 [25]	Retrospective case-control study	United States	16 patients	47.5 years	N/A
Scarlata S, 2016 [27]	Longitudinal study	Italy	81 patients	53.7 years	17.3 months
Chellapandian D, 2015 [28]	Systematic review	United States	N/A	N/A	N/A
Cheng G-S, 2015 [29]	Cohort prospective study	United States	571 patients	51. 4 years old	207 days
Choi YH, 2017 [30]	Retrospective observational study	South Korea	35 patients	10.5 years	26.1 months

Lung function decline is the first parameter to consider for developing a noninfectious pulmonary complication. Fred Hutchinson Cancer Research Center and the Seattle Cancer Alliance performed a single-center observational trial weekly for over one year, with the use of handheld spirometry in these recipients, allowing frequent testing. Clinical characteristics were labeled as: underlying disease (low, intermediate, high risk), smoking status (current, former, never, unknown) at the time of transplantation, and cGVHD (present or absent). The spirometry protocol involved patients performing handheld spirometry once before SCT and weekly for one year after SCT. The handheld spirometer only measured FEV1 and FEV6. It was compared with laboratory pulmonary function tests, showing similarity at day 80. The technique with the spirometer showed a great correlation with laboratory spirometry. It was concluded that a continuous monitorization of pulmonary function with the handheld spirometer is an alternative to laboratory-based testing for early detection of airflow decline. By the time patients get symptomatic, this indicates an advanced stage of the disease with high mortality. Pulmonary function tests are the standard exams realized to detect BOS in patients, but due to their cost and inconvenience, it makes it difficult for programs to implement [31]. 

Limitations

There were several limitations encountered while doing the research for the article. There was little information available on several pulmonary diseases, explaining the physiopathology, therapy management, risk factors, and studies conducted. Several studies were done over a long period, being affected by certain variables and sometimes only focusing on one specific diagnosis. There was scarce information on therapy management because the majority of these treatments are currently being studied and tested, so there’s no official guide to treat these complications. Current studies are being conducted that can hopefully lead to new information and discoveries for these patients. 

## Conclusions

In the last decades, new therapies have emerged to treat malignant and nonmalignant hematological diseases to improve the patient’s life prognosis. Stem cell transplantation has been modified and updated to create new techniques for different diseases and donors. Its main objective is to restore the hematopoietic function that has been eradicated by other procedures and improve the deficient immune system. Like every medical procedure, stem cell transplantation has risks and benefits that can cause severe complications or improve the patient’s actual state. These risks are mediated by several risk factors: male sex, history of pulmonary disease, and actual pulmonary condition at the time of transplant. The risk factors are greatly associated with the mortality rate and the type of complication the patient will develop. Pulmonary complications represent a total of 40%-60% of all cases secondary to SCT. Pulmonary complications include bronchiolitis obliterans syndrome, alveolar hemorrhage, fibroelastosis, pulmonary hypertension, infections, air leak syndrome, interstitial lung disease, and many other complications. The onsets of these complications are classified depending on the day of appearance and the immunocompromised state of the patient. There’s a lack of information and studies available for all these complications that dictate their physiopathology and treatment. The area of hematology is very wide, and prone to new development of treatments and procedures that could be available for new emerging diseases.
